# Positive mental health in Canadian adults who have experienced childhood sexual abuse: exploring the role of social support

**DOI:** 10.1186/s12888-022-04279-2

**Published:** 2022-10-28

**Authors:** Gillian Foley, Ken Fowler, Pam Button

**Affiliations:** 1 Department of Psychology, Faculty of Science, Memorial University of Newfoundland, St. John’s, NL Canada; 2 Student Wellness and Counselling Centre, Memorial University of Newfoundland, St. John’s, NL Canada

**Keywords:** Childhood sexual abuse, Social support, Positive mental health, Canadian adults

## Abstract

**Purpose:**

Childhood sexual abuse (CSA) is predictive of poorer mental health, greater psychiatric disorder risk, and lower positive mental health (PMH) during adulthood, outcomes potentially moderated by social support. The current study aimed to explore whether Canadian adults who have experienced CSA differ from those who have not in terms of PMH and social support. Within the CSA sample, it was further investigated whether gender differences exist with respect to PMH and social support, and if particular social support subscales predict PMH.

**Method:**

Using data from the 2012 Canadian Community Health Survey – Mental Health (CCHS-MH), 1,328 adults between 20 and 64 years reporting CSA were profiled and compared in terms of sociodemographic and socioeconomic factors, using an age, sex, and frequency matched sample of non-CSA adults. Social Provisions Scale (SPS), and the Mental Health Continuum – Short Form (MHC-SF) means were subsequently compared between the CSA and non-CSA samples, and Hierarchical regressions were conducted for CSA males and females separately to examine whether SPS subscales predicted PMH after controlling for age and income.

**Results:**

Canadian adults reporting CSA had significantly lower PMH and social support (overall and for particular subscales). For adult CSA females, guidance, social integration, and reassurance of worth predicted higher PMH, while attachment and reassurance of worth predicted higher PMH scores for CSA males.

**Conclusion:**

Adults who have experienced CSA are at risk for lower PMH and social support. Gender differences are also evident in social support subtypes that predict PMH which have important clinical implications.

## Introduction

Child sexual abuse (CSA) is defined as sexual activity perpetrated on a child which can include, but is not limited to, sexual touching, oral and/or genital penetration using a penis, fingers, or foreign objects, and/or non-contact sexual abuse such as exhibition of one’s genitals [1] achieved through manipulation, force, or abuse of power [e.g., 2, 3]. There are a range of consequences associated with having experienced CSA, including a heightened risk for developing psychiatric disorders [4–7], suicidal ideation and self-harm [4, 5, 8–11], sexual dysfunction [1, 12, 13], lower life satisfaction [5, 14, 15], and substance abuse [4, 5, 16–18].

Research suggests the consequences of CSA can have a long-lasting, deleterious impact well into adulthood [e.g., 19]. For instance, using a national sample of United States adults, Afifi et al. (16) observed a significant association between various forms of childhood maltreatment, including sexual abuse, and the risk of developing several substance use disorders [16]. Similarly, Fergusson et al.’s (4) national birth cohort New Zealand study examined associations between retrospective CSA reports and psychiatric outcomes at age 18, and found that those in the sample who experienced CSA were more likely to report higher rates of psychiatric symptoms [4]. In a follow up study, Fergusson et al. (2003) re-examined the surviving participants of the cohort used in Fergusson et al. (4) at age 30 and reported an increased risk of mental health problems, as well as decreases in self-esteem and life satisfaction, higher rates of sexual risk-taking behaviours, medical service interactions, physical illness, and welfare dependence among individuals who experienced CSA [5].

Research contends that experiencing CSA also has consequences for attachment in adulthood, potentially influencing peer, and deeper emotional relationships [20]. For instance, in their study examining the impact of various adverse childhood experiences, Cheong et al. (2017) found that adults who had experienced CSA were more likely to be divorced or separated than adults who had not had such experiences. Similarly, McCarthy and Taylor’s (1999) investigation into women reporting childhood abuse observed a relationship between such experiences and negative functioning in adult love relationships [21]. Likewise, Alexander et al. (1998) found relatively high levels of insecure relationship attachment in a sample of adult women who reported childhood incest [22].

In terms of prevalence, while reported CSA cases are typically higher among females, published rates tend to vary [23]. For example, in a systematic literature review conducted by Moody et al. (2018) featuring research between 2000 and 2017, North American CSA prevalence rates averaged 20.4% for females and 14.1% for males [24]. However, a meta-analysis of literature between 1980 and 2008 carried out by Stoltenborgh et al. (2011) estimated an average Canadian and United States CSA prevalence rate to be 20.1% for females and 8.0% for males [25]. Despite the variability, research generally does contend that CSA is underreported regardless of gender, possibly a function of victim shame, guilt, denial, as well as potential differences in CSA definitions in the literature [23, 26].

### Assessing positive mental health and social support within the context of CSA

Conceptualizations of mental health status have evolved beyond a simplistic notion of a mere absence of mental illness, to considerations more illustrative of thriving and adaptability. The World Health Organization (WHO) (2001), for example, conceives of mental health as a general state of well-being characterized by effective coping with life stressors, productive participation in society and work, and an appreciation of one’s potential [27]. Similarly, the Public Health Agency of Canada (PHAC) (2006) characterizes optimal mental health in terms of a capacity to successfully manage challenges, enjoy life, and experience sustained positive emotional, spiritual, and social well-being [28]. While the risk of compromised mental health status has been clearly linked with the experience of CSA [e.g., 19], given such an evolved multidimensional conceptualization, it is important to investigate factors that may promote instances of thriving or resilience that encourage optimal mental health.

Indeed, the literature does implicate particular factors that predict resiliency in adults who have experienced CSA including higher educational attainment and socioeconomic status, adaptive coping-skill styles, external attribution of blame, and personality attributes such as optimism or healthy emotional regulation [e.g., 29, 30]. In conceptualizing resilience, it has been conceived of as an asset acquired via dynamic, *social* interactions among individual, family, and cultural factors, despite adverse life experiences [31–35]. In this view, adaptively managing situations that could place a person at a high-risk of developing psychopathy serves to foster the development of resilience [34, 36].

Among the most empirically explored protective factors associated with resiliency within the context of CSA is social support [e.g., 29, 30, 37]. Social support may be defined as perceived or actual support from others including friends, family members, co-workers, community members  [38], involving a sense of feeling loved, cared for, belonging, or available assistance [e.g.,  39]. Research suggests that for adults with adverse childhood experiences such as abuse, neglect, and household dysfunction, salutary social support may be protective against psychiatric disorders such as depression [e.g., 20]. For instance, in an investigation exploring factors that promote resilience following CSA, a narrative review of 50 studies found that aspects of social context such as social support provided from friends, family and spouses, and involvement in social clubs or religious groups were among the strongest predictors of resiliency [37]. Similarly, a study examining resiliency in a community sample of adults who had experienced childhood physical or sexual abuse found that peer relationships in adolescence, and the quality of friendships and stability of love relationships in adulthood were strongly related to resilience [40].

Conceptually, resilience has been compared with the concept of positive mental health (PMH) [e.g.,  40] whereby Masten and Obradović (2006), for instance, define resilience as “positive patterns of adaptation in the context of adversity” [41, p. 14] with PMH representing an “individual’s ability to enjoy life, and create a balance between life activities and efforts to achieve psychological resilience” [40, p. 75]. Similar to this, and the WHO and PHAC definitions of mental health, PMH has been defined as the degree to which individuals feel a sense of control over their life, self-esteem, perceptions of personal acceptance, as well as an ability to cope with challenges [42].

Perhaps most applicable to the current study is research conducted by Bennett (2015) involving a sample of Canadian adults extracted from the 2012 Canadian Community Health Survey – Mental Health (CCHS-MH) where it was observed that adults who had experienced CSA were significantly lower in PMH than those who had not experienced CSA, and overall social support as measured by the Social Provision Scale (SPS) [38] was positively related to PMH, accounting for 25% of the variance [43].

In light of the preceding discussion, following an examination of sociodemographic and socioeconomic characteristics of Canadian adults who have experienced CSA, the current study aimed to (1) explore whether they differ from those who have not experienced CSA in terms of PMH and social support, and among those who experienced CSA, (2) assess potential gender differences with respect to PMH and social support, as well as (3) whether particular social support subscales predict PMH.

### The current study

While previous research has investigated predictors of resiliency and PMH in individuals who experienced CSA and the potential impact of social support [e.g., 43], to our knowledge, no previous research has explicitly considered PMH within the context of specific social support subtypes and gender. Therefore, the current study was designed to investigate the influence of social support (overall as well as for different subtypes) on the PMH of male and female adults who have experienced CSA using a nationally representative Canadian sample.

In line with previous research, there is reason to suspect that adults who experienced CSA will generally exhibit lower levels of both PMH and perceived social support compared with a matched sample of adults who have not experienced CSA [e.g.,  44, 45]. Moreover, it is expected that social support will positively predict PMH for adults who have experienced CSA similar to previous studies [e.g., 30]. However, since previous research has not explored whether *types* of social support predict the PMH of adults who experienced CSA, particularly within the context of gender, there were no clear expectations in this regard.

## Method

### Data Collection

Data were extracted from the Canadian Community Health Survey - Mental Health (CCHS-MH) public use microdata file [46]. The CCHS-MH is a national survey with the purpose of assessing mental health status and functioning, access to and use of mental health services and supports, and the links between mental health and covariates in Canadians. Data collection occurred between January 2nd, 2012 and December 31st, 2012 using computer-assisted personal interviewing, and while some interviews were conducted over the phone, the majority of participants were interviewed in person [46]. Residents of the three Canada territories, residents living on reserves or in Aboriginal settlements, institutionalized individuals and full-time members of the Canadian Forces were excluded from taking the survey.

As a note, readers may wonder why the older CCHS-MH (2012) data file was utilized in the current study, especially given that more recent CCHS data files exist. The simple answer is that Statistics Canada has yet to do a comprehensive follow-up ‘special mental health edition’ CCHS that contains core and essential measures vital to this study, that were administered to every respondent, representing each of the ten Canadian provinces.

### Experienced Participants

The CCHS-MH contains data representing 25,113 respondents aged 15 years or older from the ten Canadian provinces [46]. Relevant to the current study, while respondents aged 18 or over were asked questions regarding childhood maltreatment, adults aged 20 to 64 were specifically selected for the purposes of the study, resulting in a sample size of 16,972. As the CCHS-MH has been revised several times since its release, the data file used for the current study no longer captures respondents 18 and 19 years of age as discrete age categories, therefore, our adult sample included respondents aged 20 to 64. This is consistent with previous research using the CCHS-MH to explore adult respondent social support in terms of mental health issues. [47]

## Measures

### Childhood experiences of violence questionnaire – short form

For this study, in order to categorize adult respondents who experienced CSA, an item from the *Childhood Experiences of Violence Questionnaire – Short Form* was employed [48]. More specifically, while this instrument explores various physical and sexual abuse scenarios that might have occurred before 16 years of age, one primary item which seemed most direct, clear, and perhaps less vulnerable to potential memory limitations (i.e., “How many times did an adult force you or attempt to force you into any unwanted sexual activity, by threatening you, holding you down, or hurting you in some way?”) was selected. In the CCHS-MH, participants were instructed to respond to a five-point scale, ranging from “never” to “more than 10 times”, and any response other than “never” were used to assign respondents into the sample of adults who experienced CSA [48].

### The social provisions scale 10-item (SPS-10)

Social support was examined using the Social Provisions Scale 10-item (SPS-10), a ten-item questionnaire used to determine perceived social support [38]. The scale includes five types of social support including guidance (e.g., “There is someone I could talk to about important decisions in my life”), reliable alliance (e.g., “There are people I can count on in an emergency”), reassurance of worth (e.g., “I have relationships where my competence and skill are recognized”), attachment (e.g., “I feel a strong emotional bond with at least one other person”), and social integration (e.g., “I feel part of a group of people who share my attitudes and beliefs”). Each question is rated on a scale of 1 (strongly agree) to 4 (strongly disagree) whereby an overall social support score is derived using the sum of all types of social support, as well as a separate score for each of the five different types of social support. Scales were reverse coded in order for higher scores to indicate a higher level of social support and lower scores to indicate a lower level of social support [38].

### The Mental Health Continuum – Short Form (MHC-SF)

PMH was examined using the Mental Health Continuum – Short Form (MHC-SF) [49]. The instrument includes 14 questions, six questions derived from Ryff’s model of psychological well-being (e.g., “confident to think or express your own ideas and opinions”), five questions derived from Keyes’ (1998) [50] model of social well-being (e.g., “that you belonged to a community”), along with three questions assessing subjective/emotional well-being (i.e., “satisfied with life”) [42]. The instrument includes three subscales: emotional well-being, social well-being, and psychological well-being. Each item asks, “During the past month, how often did you feel…”, with responses on a six-point scale ranging from 1 (everyday) to 6 (never). Items were reverse coded and then a value of one was subtracted from each value in that “never” was given a value of 0 and “everyday” was given a value of 5. An overall score of PMH is created using the sum of the questions, with total PMH scores ranging from 0 to 70 (higher scores indicate better PMH).

### Sociodemographic and socioeconomic variables

Sociodemographic variables were categorical in nature and included gender (i.e., male, female), age (i.e., 20–34, 35–44, 45–54, 55–64), and marital status (i.e., married, common law, widowed, divorced or separated, and single), while the socioeconomic variable included personal income in Canadian dollars (i.e., less than $10,000, $10,000-$19,999, $20,000-$29,999, $30,000-$39,999, $40,000-$49,999, and $50,000 and above) [46].

### Statistical analysis

Data were analyzed using SPSS version 27. Firstly, sociodemographic and socioeconomic variables were explored via Chi Square tests to determine whether (1) the male and female CSA samples varied in terms of age grouping, and (2) marital status and income categories were dependent on whether CSA was experienced using an age, gender, and frequency matched sample of adults who had not experienced CSA.

A series of independent sample t-tests were then conducted to compare the sample of adult respondents who experienced CSA with the matched sample of adults who had not experienced CSA in terms of the average scores of PMH, and SPS-10 (overall and for each subscale). Further, for the sample of respondents who experienced CSA, independent sample t-tests were conducted to assess gender differences in PMH and SPS-10 (overall and for each subscale). Finally, two subsequent hierarchical regression analyses were carried out to explore whether, and the degree to which particular SPS-10 subscales, as well as age, and personal income, predicted PMH separately for males and females who experienced CSA.

## Results

Of the 16,972 respondents aged 20–64 years in the CCHS-MH data file, 1,328 individuals (or 7.8%) reported at least one incident whereby an adult forced (or attempted to force) them into any unwanted sexual activity before the age of 16 years (or 3.8% male and 11.3% female). For the CSA sample, Table 1 shows that a Chi Square analysis of whether age was dependent on gender was significant (χ² = 20.1, *p* < .001) in that there was a higher proportion of female adults within the younger age categories (i.e., 25.5% of females vs. 15.6% of males between 20 and 34 years of age), and a higher proportion of males in the 40-to-44-year age group (i.e., 14.6 vs. 8.8%).

**Table 1:** Gender and Age Group Percentages and Frequencies for Individuals Reporting Child Sexual Abuse (CSA) – CCHS 2012 – Mental Health

	Male	Female		
	CSA	CSA	χ2	p
	(N = 301)	(N = 1027)		
**Age (years)**			20.1	0.010
20–24	3.7(11)	8.7(89)		
25–29	5.6(17)	7.1(73)		
30–34	6.3(19)	9.7(100)		
35–39	8.6(26)	8.6(88)		
40–44	14.6(44)	8.8(91)		
45–49	11.9(36)	12.1(125)		
50–54	17.9(54)	16.1(165)		
55–59	15.9(48)	14.8(152)		
60–64	15.2(46)	14.0(144)		

As Table 2 indicates, marital status was significantly dependent on whether CSA was experienced, and the trend was quite similar for males and females (χ² = 18.45, *p* < .001 and χ² = 36.3, *p* < .001 respectively). For instance, compared to the matched sample of non-CSA adults, males and females reporting CSA were less likely to be married (36.5 vs. 51.8% and 35.1 vs. 47.2% respectively), and more likely to be single (31.2 vs. 25.9% and 26.9 vs. 22.4% respectively). However, the table also suggests that while males reporting CSA were more likely to be separated or divorced (16.6 vs. 14.0%), this trend was more pronounced for females who experienced CSA (21.4 vs. 14.6%).

**Table 2:** Marital Status and Personal Income Percentages and Frequencies by Gender for Adults Reporting Child Sexual Abuse (CSA) and an Age, Gender and Frequency Matched Non-CSA Sample – CCHS 2012 – Mental Health

		Percent (N)							
		**Male**				**Female**			
		CSA	Non-CSA	χ2	p	CSA	Non-CSA	χ2	p
		(N = 301)	(N = 301)			(N = 1027)	(N = 1027)		
**Marital Status**									
	Married	36.5(110)	51.8(156)	18.45	0.001	35.1(358)	47.2(485)	36.3	0.000
	Common-Law	14.0(42)	8.3(25)			13.5(137)	12.1(125)		
	Widowed	1.3(4)	0(0)			3.1(32)	3.5(36)		
	Divorced/Separated	16.6(50)	14.0(42)			21.4(218)	14.6(150)		
	Single	31.2(94)	25.9(78)			26.9(274)	22.4(230)		
**Personal Income**									
	< $10,000	2.4(7)	1.7(5)	38.9	0.000	8.3(86)	7.7(72)	75.7	0.000
	$10,000-$19,999	19.6(57)	6.1(17)			25.5(247)	12.9(121)		
	$20,000-$29,999	21.6(63)	14.3(40)			27.9(270)	25.1(235)		
	$30,000-$39,999	12.4(36)	10.7(30)			11.7(113)	14.2(133)		
	$40,000-$49,999	11.3(33)	15.1(42)			8.2(80)	9.7(91)		
	$50,000 +	32.7(95)	52.0(145)			17.8(172)	30.3(283)		

Table 2 also shows that personal income was significantly dependent on whether CSA was experienced, with a similar trend evident for males and females (χ² = 38.9, *p* < .001 and χ² = 75.7, *p* < .001 respectively). However, while 43.7% of males who experienced CSA earned less than $30,000 (compared to 22% of the matched non-CSA sample), 65.1% of females who experienced CSA earned less than $30,000 (compared to 52.2% of the matched sample).

Table 3 presents the means, standard deviations, *t*-values, *df*, and Cohen’s *d* associated with PMH, the SPS-10, and each of the SPS-10 subscales for those reporting CSA and those not reporting CSA. Tests of 14 a priori hypotheses were assessed using two independent groups t-tests with a Bonferroni adjusted alpha level of 0.003 per test (0.05/14). As the table shows, the PMH mean for adults who experienced CSA was significantly lower than that of the matched, non-CSA sample mean (i.e., *M*_*CSA*_ = 48.46 vs. *M*_*Non−CSA*_ = 53.67), *t*(2470) = 10.38, *p* < .001 respectively). The table also shows that SPS-10 overall mean, and that of each of the five subscales (i.e., attachment, social integration, guidance, reassurance of worth, and reliable alliance) were significantly lower for the CSA adult sample compared to the matched, non-CSA adult sample.

**Table 3:** Means, Standard Deviations, t values, df, and Cohen’s d associated with PMH and SPS-10 Subscales for CSA Sample and Non-CSA Matched Sample

	Non CSA Matched Sample				CSA Sample				
		N = 1,328		N = 1,328					
		*M*	*SD*	*M*		*SD*	*t*	*df*	Cohen’s *d*
Positive Mental Health		53.67	11.20	48.46		13.67	10.38***	2470	0.41
Social Provisions Scale		36.03	4.29	34.73		5.30	6.92***	2608	0.27
Attachment		7.29	1.00	7.04		1.21	5.97***	2649	0.23
Guidance		7.31	1.00	7.06		1.27	5.83***	2651	0.23
Reliable alliance		7.39	0.86	7.11		1.20	6.86***	2652	0.27
Social integration		6.98	1.11	6.62		1.34	7.54***	2641	0.29
Reassurance of worth		7.01	1.00	6.80		1.20	4.87***	2625	0.19

***p < .001

Table 4 presents the PMH and SPS-10 means, standard deviations, *t*-values, *df*, and Cohen’s *d* for the male and female Canadian adults reporting CSA. As the table shows, the PMH mean for CSA males did not differ from that of the CSA females (*M*_*Male*_ = 48.29 vs. *M*_*Female*_ = 48.51), *t*(1238) = -0.24, *p* = .811. The table also shows that SPS-10 overall mean, and that of attachment and social integration were significantly lower for the CSA males based on the Bonferroni adjusted alpha level of 0.003.

**Table 4:** Means, Standard Deviations, t values, df, and Cohen’s d associated with PMH and SPS-10 Subscales for the CSA Male and Female Sample

	CSA Male Sample			CSA Female Sample				
		N = 301		N = 1,027				
		*M*	*SD*	*M*	*SD*	*t*	*df*	Cohen’s *d*
Positive Mental Health		48.29	14.85	48.51	13.32	-0.24	1238	0.02
Social Provisions Scale		33.55	5.68	35.07	5.13	-4.35***	1298	0.29
Attachment		6.78	1.35	7.11	1.15	-4.26***	1324	0.28
Guidance		6.84	1.29	7.12	1.26	-3.32**	1324	0.22
Reliable alliance		6.93	1.24	7.16	1.18	-3.00**	1326	0.20
Social integration		6.37	1.32	6.69	1.34	-3.56***	1316	0.23
Reassurance of worth		6.63	1.33	6.85	1.15	-2.78*	1309	0.18

*p < .05, **p < .005, ***p < .001

A separate hierarchical regression was conducted to determine whether, and degree to which specific SPS-10 subscales predicted PMH for Canadian *male*s who experienced CSA after controlling for age and personal income in block 1 (See Table 5 for a summary). Accordingly, block 1 of the analysis accounted for 11.3% of the variation, with personal income significantly and positively predicting PMH (*t*(264) = 5.95, *p* < .001 respectively). Introducing the SPS-10 subscales in block 2 accounted for an additional 33.3% of the variation, with attachment and reassurance of worth emerging as significant and positive predictors [*t*(259) = 3.25, *p* < .001, and *t*(259) = 2.29, *p* = .023 respectively].

**Table 5:** Summary of Hierarchical Regression Analysis of Variables Predicting PMH in the Male CSA Sample

	Model 1				Model 2		
Predicator Variable	*B*	*SE*	*β*	*B*		*SE*	*β*
Age Total personal income *Social Support* Attachment Guidance Reliable alliance Social integration Reassurance of worth *Adjusted R* ^*2*^ *F* for *R*^*2*^ change	0.15 3.18	0.38 0.53 0.113 17.889***	0.02 0.34***	-0.21 1.47 2.96 0.86 0.37 1.51 1.87		0.31 0.45 0.91 0.91 0.95 0.85 0.82 0.446 31.580***	− 0.03 0.16** 0.28** 0.08 − 0.03 0.14 0.17*

Note: N = 301

*p < .05, **p < .005, ***p < .001

A subsequent hierarchical regression was used to determine whether, and degree to which specific subscales of SPS-10 predicted PMH for Canadian *females* who experienced CSA after controlling for age and personal income in block 1 (See Table 6 for a summary). Block 1 accounted for 4.3% of the variation with personal income significantly and positively predicting PMH (*t*(891) = 6.22, *p* < .001). Introducing the SPS-10 subscales in block 2 accounted for an additional 31.7% of the variation of PMH, with guidance, social integration and reassurance of worth significantly and positively predicting PMH [*t*(886) = 3.16, *p* = .002, *t*(886) = 7.92, *p* < .001, and *t*(886) = 6.01, *p* < .001, respectively].

**Table 6:** Summary of Hierarchical Regression Analysis of Variables Predicting PMH in the Female CSA Sample

	Model 1				Model 2		
Predicator Variable	*B*	*SE*	*β*	*B*		*SE*	*β*
Age Total personal income *Social Support* Attachment Guidance Reliable alliance Social integration Reassurance of worth *Adjusted R* ^*2*^ *F* for *R*^*2*^ change	0.24 1.72	0.17 0.28 0.043 21.299***	0.05 0.20***	0.40 0.57 -0.37 1.61 -0.62 3.35 2.77		0.14 0.23 0.60 0.51 0.50 0.44 0.46 0.360 72.871***	0.08* 0.07* − 0.03 0.15** − 0.05 0.34*** 0.24***

Note: N = 1,027

*p < .05, **p < .005, ***p < .001

## Discussion

Overall, it was observed that 7.8% of Canadian adults between 20 and 64 years reported CSA. Compared with other prevalence rates reported in the literature [e.g., 23, 24], this rate appears lower, perhaps the result of a narrower sample age-range utilized in this study which excluded respondents between 17 and 19 years, and those greater than 64 years. Further, as acknowledged in other studies [e.g., 23, 26], the CSA definition may have also yielded a lower rate since it was specifically predicated on ‘forced (or attempted to force) sexual activity’, that was perpetrated by an adult (as opposed to another child or youth). Despite a relatively conservative overall prevalence, however, the rate observed for Canadian females was higher than males (i.e., 11.3 vs. 3.8% respectively), a trend consistently reported in previous studies [e.g., 23–25].

### Sociodemographic and socioeconomic findings

In terms of age, it was observed that the female CSA sample was younger, with approximately one quarter between 20 and 34 years. Interestingly, while the proportions reporting being ‘married’ or ‘common law’ were comparable between CSA males and females, CSA females had a higher proportion reporting being divorce or separated, while CSA males were more likely to report being single.

An analysis of personal income statistics revealed that CSA respondents were more likely to report lower incomes than non-CSA respondents, a finding consistent with previous research observing higher rates of welfare dependence among individuals who have experienced CSA [e.g., 5]. However, it was further observed that this trend was notably dependent on gender as approximately 65% of CSA female respondents were more likely to earn less of than $30,000 compared to 44% in the CSA male sample.

### Social support and PMH findings

A primary goal of the present study was to examine the role of social support in the relationship between CSA and PMH. To our knowledge, this is one of only two studies examining the PMH of adults who have experienced CSA, and as predicted, both levels of PMH and perceived social support were lower for adults who experienced CSA compared to means of a matched, non-CSA adult sample. Further, our expectation that higher levels of overall social support would predict higher levels of PMH for the adults who experienced CSA was also supported. While there were no predictions made about the types of social support that might positively predict PMH for adults who experienced CSA, and between the males and females who experienced CSA, there were some interesting findings.

In terms of PMH, while the current study found that scores were significantly lower for adults reporting CSA, they did not differ between males and females within this sample, results that seem to suggest that males and females might be equally impacted by the traumatic experiences of CSA in terms of lower PMH. Interestingly, however, while a subsequent comparison between 20 and 64 year-old Canadian adults from the CCHS-MH [46] who had *not* experienced CSA revealed significantly higher PMH for women, significantly higher distress levels, and greater proportions reporting depression and anxiety were also observed, findings that appear in line with previous research. For instance, Collishaw et al. [44] found that there is in an increased risk for females developing mental health problems in adulthood, while poor mental health outcomes in adulthood following CSA are equally likely between males and females [5]. For such observations, however, the obvious question is how it might be possible that higher psychological distress and incidences of depression and anxiety can coexist with superior PMH for women who had *not* experienced CSA compared to men who had *not* experienced CSA? While such is beyond the scope of the current paper, perhaps the key lies within the multi-dimensional character of PMH which captures not only psychological well-being, but also social and subjective/emotional well-being [42]. Perhaps individuals can exhibit resilience in terms of social well-being, but be somewhat compromised in terms of emotional or psychological wellness.

When examining perceived levels of social support, subscale and overall SPS-10 scores were significantly lower for adults who experienced CSA when compared to the means of the matched, non-CSA adult sample. These results provide corroborating evidence for previous research looking at social support following CSA [e.g.,  44, 45]. Golding et al. (2002), for example, found that a sexual assault history in both childhood and adulthood were related to low levels of social support compared to those who were not sexually assaulted including, being less likely to be married, receiving less emotional support from others, and less frequent contact with others [45]. Similarly, Collishaw et al. found that almost half of the adolescents in their sample who experienced CSA showed abnormalities in their interactions with peers in terms of low peer acceptance, and poorer friendship quality [44].

When comparing the male and female adults who experienced CSA on their level of overall social support and SPS-10 subscales, females had significantly higher levels for social support outcomes than the males, particularly for attachment and social integration. This observation could be reflected in previous research which has observed that females who experienced CSA tend to receive more positive social reactions to abuse disclosure than males who experienced CSA, and that females have higher rates of CSA disclosure than males [51]. Further, since males are typically socialized to be less expressive, perhaps reporting CSA would contradict such expectations [52], potentially resulting in relatively lower perceptions of social support. In terms of sociodemographic statistics, recall that the female CSA sample had a younger age profile, a finding that may indicate a delay in the degree to which male CSA respondents choose to acknowledge, and hence report such childhood trauma. Taken together, such findings clearly indicate that more in-depth assessment is warranted in understanding potential gender differences in social support for adults who have experienced CSA.

While there were no predictions in terms of the types of social support that might significantly predict PMH, hierarchical regression analyses featuring females who have experienced CSA revealed that guidance, social integration, and reassurance of worth were positively associated with PMH after controlling for age and income. Interestingly, however, a subsequent hierarchical regression revealed that while reassurance of worth also emerged as a significant predictor of PMH for adult males who have experienced CSA, guidance was not significant, but attachment was. These results suggest that although the potential salutary influence of social support exists for both men and women in predicting higher PMH, males and females who experienced CSA seem to differ in the types of social support that are most beneficial. For males who have experienced CSA, for instance, it would make sense that attachment emerged as research does suggest that a history of childhood maltreatment may result in a detriment in the capacity to form attachments or alliances with others in adulthood with the severe erosion of trust [e.g.,  53-57]. Also as a corroborating point of interest, recall that a higher proportion of CSA males reported being single compared to CSA females.

For females who experienced CSA, however, it would appear that social support in the form of social integration, and guidance or advice offered by others seem to have more salutary benefit. Differences in social support for males and females who experienced CSA may also be reflected in marital status, specifically with respect to higher rates of separation and divorce for women. As women who have experienced CSA are at a high risk for dysfunctional relationships and intimate partner violence [58], perhaps women are more likely to leave intimate relationships as they show lower levels of attachment and instead have support in the form of guidance from others. Without question, future research exploring the nature of perceived social support and social context could provide valuable insights/information that could inform clinical interventions, service provision, and support.

### Limitations and future directions

It is important to mention the limitations of the present study. One limitation includes the fact that this research was correlational. While the relationship between CSA and PMH was significant, it cannot be concluded that a history of CSA causes lower levels of PMH, nor that certain types of social support cause higher levels of PMH. Another limitation involves the use of a secondary data set. There are other variables that impact the outcomes of CSA, such as age of onset, chronicity, and perpetrator characteristics [37], that could not be examined as they were not captured in the CCHS-MH. Similarly, the CCHS-MH excluded incarcerated individuals and those living on reserves or Aboriginal settlements from their sample, two populations that have a high rate of CSA prevalence [56, 57]. Future research should examine the variables of CSA using a more inclusive population sample to further determine the factors, including social support, that impact PMH. An additional limitation to the present study was the definition of CSA being used. While the definition of CSA was a severe form of CSA, it did not capture different levels of severity which have been previously shown as an important factor in the likelihood of developing resilience [5]. However, it can be assumed given the severity of the definition that the experience was quite traumatic. Future studies should replicate this study using a broader definition of CSA to understand the impact of the severity and characteristics of the abuse. Related, there is a lack of international classification for sexual violence and CSA which must be addressed. As long as there remains no such classification, it will be difficult to accurately compare studies and international statistics based on CSA data. Although the study data for the current study were not collected during COVID-19 and could not be addressed, we believe it is important for future research to examine the impacts COVID-19 has had on the relationship between social support, PMH and CSA. Lastly, like previous research examining CSA [e.g., 44], we expect that CSA was underreported in the CCHS-MH dataset. However, while there is no way to determine the rate of false negatives in our sample, previous research suggests that the risks of psychiatric outcomes are robust despite reporting errors [ 59].

## Conclusion

The current study adds to a very limited body of research exploring the PMH of adults who have experienced CSA, and the only known study to examine an association between *types* of social support and the PMH within the context of gender. The use of the CCHS-MH dataset allowed for an investigation of a large, representative population sample of Canadian adults who experienced CSA whereas the majority of research examining the impacts of CSA in adulthood examine clinical samples, thus this research is unique and important to understanding CSA in Canada. The findings of the present study suggest that particular types of social support are important for the PMH of males and females who have experienced CSA, and thus may have significant clinical implications.

## Data Availability

Please check the submission guidelines:https://bmcpsychiatry.biomedcentral.com/submission-guidelines/preparing-your-manuscript/research-article.

## Declarations

Hence, this is to confirm that all methods were carried out in accordance with relevant guidelines and regulations.
